# The effect of *Euphorbia szovitsii* Fisch. & C.A.Mey extract on the viability and the proliferation of MDA-MB-231 cell line

**DOI:** 10.1042/BSR20181538

**Published:** 2019-01-11

**Authors:** Majid Asadi-Samani, Mahmoud Rafieian-Kopaei, Zahra Lorigooini, Hedayatollah Shirzad

**Affiliations:** 1 Medical Plants Research Center, Basic Health Sciences Institute, Shahrekord University of Medical Sciences, Shahrekord, Iran; 2 Cellular and Molecular Research Center, Basic Health Sciences Institute, Shahrekord University of Medical Sciences, Shahrekord, Iran

**Keywords:** Apoptosis, Cell cycle analysis, Drug discovery, Medicinal plants, Toxicity

## Abstract

Some medicinal herbs and compounds are known to target cancer cells, but the success of them as anticancer compounds depends to a large extent on their ability to activate pathways that kill cancer cells by arresting cell cycle and inducing apoptosis. The aim of the present study was to determine the anticancer effects of *Euphorbia szovitsii Fisch. & C.A.Mey.* on the breast cancer cells to reveal the underlying mechanism of its anti-breast cancer properties. In this experimental study, triple negative breast cancer cell line (MDA-MB-231) was cultivated in RPMI-1640 medium. Hydroalcoholic extract (70:30) of aerial parts of the plant was prepared. The cultured cells were treated with different concentrations (0–1000 μg/ml) of *E. szovitsii* extract for 24 and 48 h. Toxicity of the extract on MDA-MB-231 cells was examined using MTT (3-[4,5-dimethyl-2-thiazolyl]-2, 5 diphenyl tetrazolium bromide) test. The Annexin V–FITC Apoptosis Detection Kit was used to evaluate apoptosis and necrosis. Flow cytometry technique was employed to differentiate different phases of the cell cycle in the cells. Data were analyzed by GraphPad Prism and SPSS software. After 24 and 48 h, the IC50 values were respectively 76.78 (95% CI = 60.75–97.05; *R* = 0.8588) and 59.71 (95% CI = 46.25–77.09; *R* = 0.8543) μg/ml for *E. szovitsii*. The extract exhibited antiproliferative effects against MDA-MB-231 cells in a dose-dependent manner. Annexin V-FITC/PI assay confirmed that the extract was able to induce apoptosis in MDA-MB-231 cells. Moreover, treatment with the extract resulted in cell cycle arrest at G1 phase. Therefore, *E. szovitsii* could induce apoptosis and cycle arrest in the MDA-MB-231 cell line. It might be a good resource of natural products for producing anti-breast cancer drugs.

## Introduction

Breast cancer is the most common cancer in women around the world. It is the leading cause of cancer death in less developed countries. According to the global cancer project (2012), nearly 1.7 million new cases of breast cancer have been diagnosed (12% of all new cancer cases) and 521,907 cases of deaths due to breast cancer occurred in the world [1,2].

Despite the many drugs used to treat breast cancer, the incidence and prevalence of this cancer is still high. Besides that, many of the chemical drugs used to treat breast cancer lead to several side effects that limit their use for patients. Meanwhile, the production of drugs with higher efficacy and fewer side effects have always been taken into account. Medicinal plants are one of the most important natural resources of the options offered by researchers and pharmacists and, if they are used at certain doses, they will show the highest compatibility with the immune system. Medicinal plants, in many cases, have antioxidant and anti-inflammatory properties that can reduce the side effects of chemical drugs [3,4]. In previous studies on the anticancer effects of medicinal plants in Chaharmahal and Bakhtiari province, a plant from the Euphorbia family, namely, *E. szovitsii* Fisch. & C.A.Mey., was among the plants that exhibited anticancer effects on various cancers such as breast cancer. In addition, in examining cells under treatment, significant morphological changes were observed, including major changes in the normal state of cell membrane and cell granulation, shrinkage of the membrane of the nucleus, and decrease in the cell volume. In addition, a number of cells have been isolated from the flask floor and floated.

In this paper, we have evaluated the anticancer effects from *E. szovitsii* on the MDA-MB-231 cells to determine the underlying mechanism of its anticancer effects.

## Experimental

### Cell culture

Triple negative breast cancer cell line (MDA-MB-231 cells) was purchased from Pasteur Institute, Iran-Tehran. The cells were grown in RPMI-1640 medium at 37°C in a humidified atmosphere with 5% CO_2_, 95% air. The medium was refreshed every 24 h, and the cells were trypsinized (0.1% trypsin) on reaching 80% confluency.

### Preparation of the herbal extract

*E. szovitsii* was collected from Saman in Chaharmahal and Bakhtiyari province and air dried in the shade at RT (25°C). The plant was authenticated by Dr Shirmardi (Research Center for Agricultural & Natural Resources, Shahrekord, Iran). A voucher specimen was prepared and deposited in Herbarium unit of Shahrekord University of Medical Sciences (Skums-935). The aerial parts were ground by a mechanical grinder. The hydroalcoholic extract (alcohol:water = 70:30) of the plant was provided using maceration method at room temperature. The extract was then filtered through Whatman paper no. 40, and the resultant filtrate was evaporated under negative pressure using a rotary vacuum evaporator.

### Cell viability assay

For evaluating the cell viability, MDA-MB231 cells were exposed to a wide concentration range of the *E. szovitsii* extract (0–1000 µg/ml). Toxicity of the extract on breast cancer cells was examined using MTT (3-[4,5-dimethyl-2-thiazolyl]-2, 5 diphenyl tetrazolium bromide) assay 24 and 48 h after seeding.

Briefly, 7 × 10^3^ cells were seeded into each well of a 96-well plate. After 24 h, the attached cells were treated with different concentrations (0–1000 μg/ml) of *E. szovitsii* extract in two time intervals (24 and 48 h). The negative control was not treated with any concentration of *E. szovitsii* extract. After incubation, supernatant was discarded and the attached cells rinsed with PBS. Then, 100 MTT solution (2 mg/ml PBS) was added to each well and incubated 4 h at 37°C. Eventually, supernatant of each well was removed again, mixed with 200 μl of DMSO and gently shaked for a while to subsequently keep in dark place.

The cell viability of the treated cells was measured at 570 nm by Stat-Fax 2100 microplate reader (Awareness Technology Inc) and the calculated according to the following equation:

$$\text{Percentage of Viability}=\frac{\text{proportion of sample absorbance}}{\text{control absorbance}}.$$

### Apoptosis analysis

For evaluating the apoptosis assay, the flow cytometry technique was used. MDA-MB231 cells were seeded in six-well plates at a density of approximately 120 × 10^3^ cells. Twenty-four hours after seeding, the cells were exposed with IC50 values of *E. szovitsii* extract. The treated cells were harvested after 24 and 48 h, rinsed twice with cold PBS. The pellet (at a concentration of 10^6^ cells/ml) resuspended in 500 μl Binding Buffer (1×), and incubated with 5 μl FITC-Annexin V and 5 μl PI. The samples were gently vortexed and incubated for 15 min at room temperature in the dark. About 400 µl of 1× Binding Buffer was added to each tube [5]. One hour after incubation, flow cytometric technique was performed by a flow cytometer (CyFlow, Partec, Munster, Germany). The results were analyzed using FCS Express 5 reader (De Novo Software).

### Cell cycle assay

For calculating the content of DNA in various phases of cell cycle, the cells were seeded into six-well plate and treated with IC50 values of *E. szovitsii* extract 24 h after seeding. Cell cycle assay was conducted according to the standard protocol 24 and 48 h after treatment. Briefly, 5 × 10^5^ treated cells were incubated with 250 μl of trypsin buffer (as Solution A) and 200 μl of trypsin inhibitor with RNase buffer (as Solution B) at 20–25°C for 10 min, respectively. Then, 200 μl of cold PI stain solution (as Solution C) was added and incubated in the dark for 10 min on ice [6]. Finally, the samples were read via a flow cytometer (CyFlow, Partec, Munster, Germany), and data were analyzed through FCS Express 5 reader (De Novo Software).

### Statistical analysis

The IC50 values were determined using GraphPad Prism 6.0 software through regression analysis. ANOVA (one-way analysis of variance) was used to compare the data. *P*<0.05 was considered significant.

## Results

**Figure 1:**
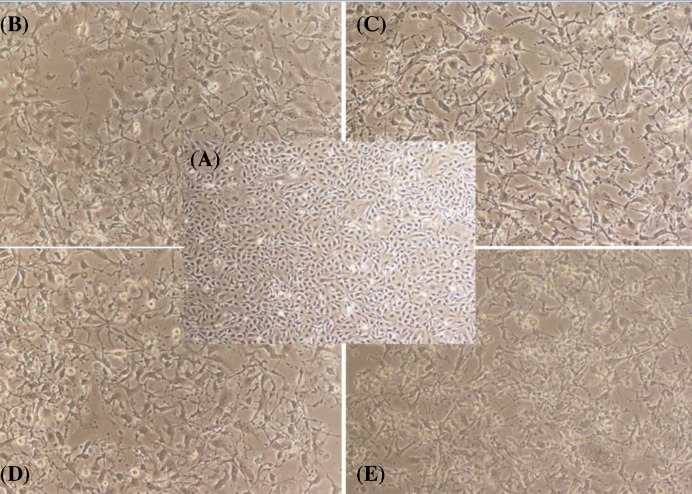
Microscopic photos of triple negative breast cancer cell line (MDA-MB231) treated with the *Euphorbia szovitsii* Fisch. & C.A.Mey. extract (**A**) Control cells (none treated cells); (**B**) Treated by 50 µg/ml of *E. szovitsii* after 24 h; (**C**) Treated by 50 µg/ml of *E. szovitsii* after 48 h; (**D**) Treated by 100 µg/ml of *E. szovitsii* after 24 h; (**E**) Treated by 100 µg/ml of *E. szovitsii* after 48 h.

**Figure 2:**
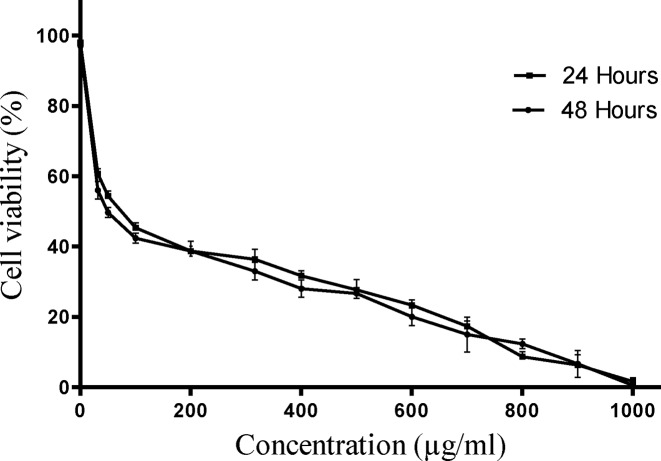
Cell viability evaluation of triple negative breast cancer cell line (MDA-MB231) treated with the *Euphorbia szovitsii* Fisch. & C.A.Mey. hydroalcoholic extract

*E. szovitsii* extract indicated cytotoxic effects on MDA-MB231 cells obviously. The extract exhibited cytotoxic effects on breast cancer cell line in a dose-dependent manner (Figure 1). The IC50 values were gained 76.78 (95% CI = 60.75–97.05; *R* = 0.8588) and 59.71 (95% CI = 46.25–77.09; *R* = 0.8543) μg/ml 24 and 48 h after treatment, respectively (Figure 2).

### *E. szovitsii* induced apoptosis in breast cancer cell line

**Figure 3:**
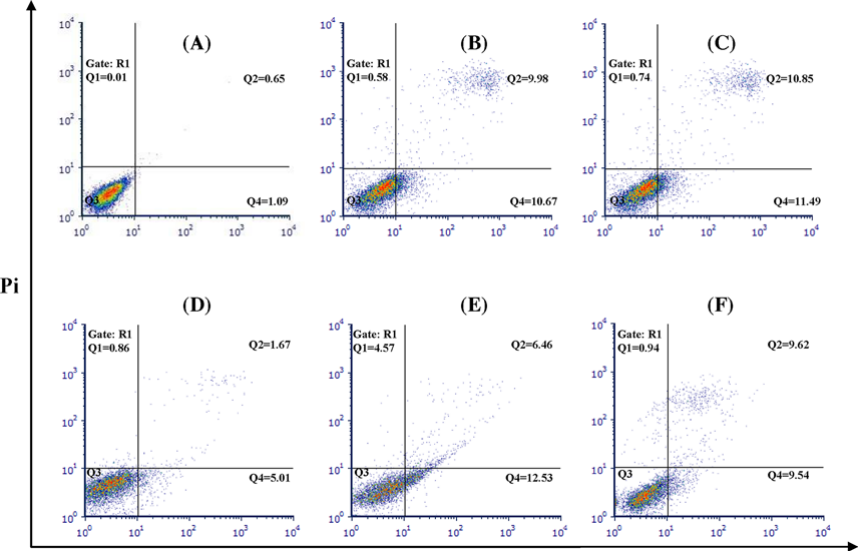
*Euphorbia szovitsii* Fisch. & C.A.Mey. hydroalcoholic extract induces apoptosis in the MDA-MB231 cells (**A**) Control cells (none treated cells) after 24 h; (**B**) Treated by 50 µg/ml of *E. szovitsii* after 24 h; (**C**) Treated by 100 µg/ml of *E. szovitsii* after 24 h; (**D**) Control cells (none treated cells) after 48 h; (**E**) Treated by 50 µg/ml of *E. szovitsii* after 48 h; (**F**) Treated by 100 µg/ml of *E. szovitsii* after 48 h. Q4: early apoptotic cells (Annexin V-FITC positive, PI negative); Q2: late apoptotic cells (Annexin V-FITC positive, PI positive); Q1: necrotic cells (Annexin V-FITC negative, PI positive).

In order to apoptosis evaluation, MDA-MB231 cells were treated with 50 (as minimum concentration of IC50 value) and 100 µg/ml (as maximum concentration of IC50 value) of *E. szovitsii* extract. Flow cytometry technique via FITC Annexin V Apoptosis Detection Kit II was used to determine necrotic and apoptotic cells. In this method, Annexin V-FITC positive and PI negative cells were considered as early apoptotic cells (Q4); Annexin V-FITC positive and PI positive cells were considered as late apoptotic cells (Q2); and Annexin V-FITC negative and PI positive cells were considered as necrotic cells (Q1) (Figure 3).

**Figure 4:**
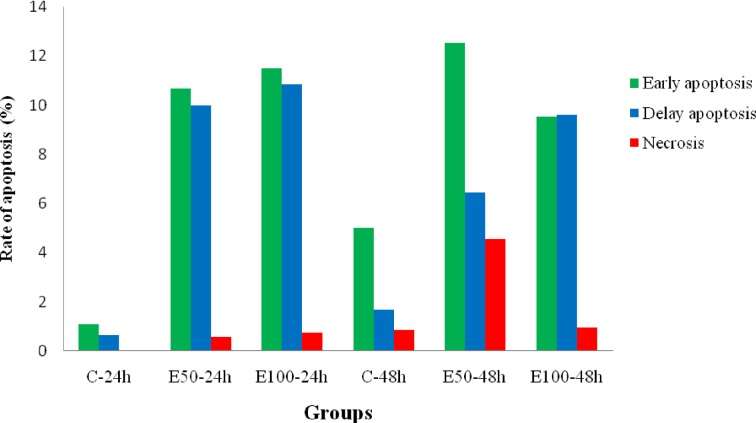
The rate of apoptosis in treated MDA-MB231 cells with *Euphorbia szovitsii* Fisch. & C.A.Mey. hydroalcoholic extract C: Control; E50: *E. szovitsii* with 50 µg/ml concentration; E100: *E. szovitsii* with 100 µg/ml concentration.

Sum of early apoptotic cells and late apoptotic cells was calculated as the rate of apoptosis. The results indicated an increase in apoptotic cells in 24 and 48 h after treatment via IC50 values of the *E. szovitsii* extract (Figure 4).

### *E. szovitsii* arrested cell cycle in breast cancer cell line

**Figure 5:**
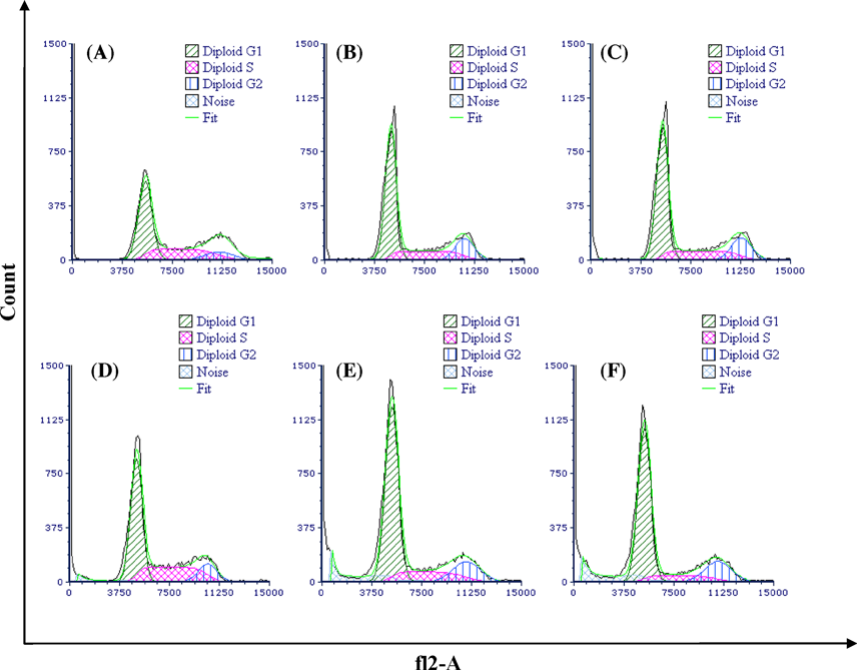
*Euphorbia szovitsii* Fisch. & C.A.Mey. hydroalcoholic extract arrest cell cycle in the MDA-MB231 cells (**A**) Control cells (none treated cells) after 24 h; (**B**) Treated by 50 µg/ml of *E. szovitsii* after 24 h; (**C**) Treated by 100 µg/ml of *E. szovitsii* after 24 h; (**D**) Control cells (none treated cells) after 48 h; (**E**) Treated by 50 µg/ml of *E. szovitsii* after 48 h; (**F**) Treated by 100 µg/ml of *E. szovitsii* after 48 h.

DNA content was analyzed in different phases of the cell cycle and percentage of the cells in any phases was compared with the control groups (Figure 5). Treatment of breast cancer cell line with *E. szovitsii* extract caused an increase in the percentage of the cells at the G1 phase; whereas a decrease was seen in the cell population at the S phase. G2 phase indicated a decrease until 48 h, but after 48 h it indicated an increase in proportion compared with the control cells. The cells treated with 50 and 100 µg/ml of *E. szovitsii* after 48 h showed a decrease in proportion of the cells in G2/M (Table 1). Percentage of the cells in different phases of the cell cycle in treated cells was significantly different from them in the control group (*P*<0.05).

**Table 1:** Percentage of the MDA-MB231 cells treated with *Euphorbia szovitsii Fisch. & C.A.Mey.* hydroalcoholic extract in different phases of the cell cycle

Groups		G1 (%)	S (%)	G2 (%)	G2/G1
24 h	Control	43.23	39.06	17.71	1.92
	E50	58.25	30.01	11.74	1.91
	E100	62.09	22.78	15.14	1.93
48 h	Control	54.35	36.32	9.33	1.98
	E50	67.58	18.89	13.54	1.92
	E100	69.18	13.38	17.44	1.89

*E50, E. szovitsii* with 50 µg/ml concentration; *E100, E. szovitsii* with 100 µg/ml concentration.

## Discussion

In present study that designed to evaluate the anticancer effects of *E. szovitsii* on the MDA-MB-231 cells and to determine the underlying mechanism of its anticancer effects, the studied plant could induce apoptosis and arrest cell cycle in the breast cancer cell line.

Today, one of the interesting strategies that has been considered in cancer chemotherapy is drug interventions that can mediate the death of malignant cells by inducing apoptosis. In the present study, the extract of *E. szovitsii* both higher and lower than IC50 concentrations, was able to kill the cancer cells by inducing apoptosis. The plant was able to exhibit its apoptotic effect even after 24 h, and there was no significant difference in apoptosis between 24 and 48 h after treatment. So far, many medicinal plants and compounds have been observed to show anticancer effects by inducing apoptosis. Although many plants are able to induce apoptosis, in some studies, the extracts have been observed to induce death through the necrosis pathway, which limits their use to some extent. In addition, most of the plants evaluated in the previous studies have shown greater apoptotic effects after 48 h [7–11]. Based on the results of the present and previous studies, this plant may be considered as one of the potential anticancer plants due to its anti-apoptotic effects after 24 h, as well as the low rate of necrosis induction.

In cancer cells, the regulation of the cell cycle control points was disturbed, resulting in a decrease in the percentage of cells, in particular the cells present in the G1/G0 phase, and the percentage of the cells present in the S phase [12]. In our study, the anticancer effect of the extract on cell cycle phases may suggest that the extract at both lower and higher than IC50 levels can arrest the cell cycle in the G1 phase and also decrease the amount of cells in the S phase. In the study of Choene et al., the growth inhibiting effects of the *E. turicalli* extract on different breast cell lines including MDA-MB 231 and MCF-7 were investigated. The plant extract exhibited growth inhibiting effects in cell type- and concentration-dependent manner after 48 h and arrested the cell cycle in the G0/G1 phase due to excessive expression of p21 [13]. Studies have shown plants that are selectively able to arrest the cell cycle in a particular phase can be a good choice for anticancer therapies, and compounds that arrest the cell cycle during the G1/S transitional phase are typically nucleotide synthesis inhibitors [14]. These results indicate that the plant extract, as a nucleotide synthesis inhibitor, can be a good choice to develop anticancer drugs especially to treat breast cancer. In the study of Choene and Motadi, various types of secondary metabolites including flavonoids and terpenes in *E. tirucalli* were reported, and the anticancer effects of this plant were attributed to these compounds [13].

In Iran, Zare et al. investigated the chemical composition of another species of the Euphorbia genus, namely, *E. macrostegia*, and observed that the plant had a wide range of active compounds, including tertipanes and unsaturated fatty acids, which can have anticancer effects [15]. Specifically, the anticancer effects of other compounds such as jolkinolide B, euphoscopin, euphohelioscopin, euphornin A, helioscopinolide, steroids and triterpenes obtained from different species of the Euphorbia genus have been studied on various breast cancer cells and their positive effects have been confirmed [16–19]. The results of the present and previous studies indicate that the species of the Euphorbia genus may be able to exert anticancer effects due to the presence of terpenoid compounds.

## Conclusion

*E. szovitsii* indicated anti-breast cancer effects with visible morphological changes on the MDA-MB231 cell line. The extract could induce apoptosis and cell cycle arrest in the MDA-MB-231 cell line. We believe that *E. szovitsii* extract can be considered as a proper candidate for cancer therapy. It should be evaluated in *in vivo* studies and human clinical trials for producing an anti-breast cancer drug.

## Abbreviations

C: Control
E.: Euphorbia
E50: E. szovitsii Fisch. & C.A.Mey. with 50 µg/ml concentration
E100: E. szovitsii Fisch. & C.A.Mey. with 100 µg/ml concentration
RT: room temperature
